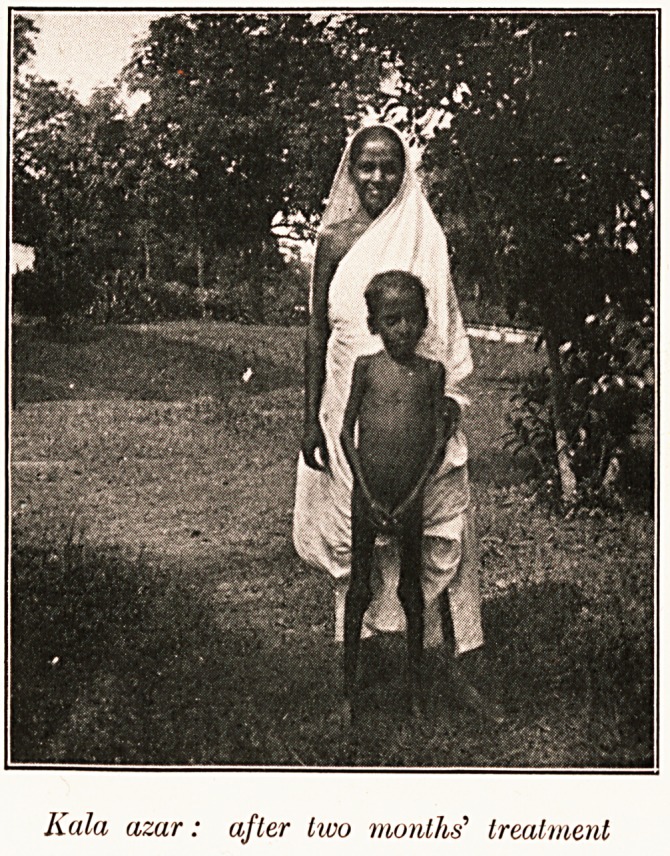# Kala Azar. Notes on the Disease and Its Treatment

**Published:** 1926

**Authors:** Erskine Faraker

**Affiliations:** (Keinton Mandeville), Late Cheif Medical Officer, Mission Hospital, Kalna, Burdwan, Bengal


PLATE XII.

*Kala azar: before treatment.*

*Kala azar: after two months' treatment*

### The Bristol Medico-Chirurgical Journal

" *Scire est nescire, nisi id me*
*Scire alius sciret.*"

WINTER, 1926.

### KALA AZAR.

### NOTES ON THE DISEASE AND ITS TREATMENT.

BY

ERSKINE FARAKER, M.R.C.S., L.R.C.P.
(*Keinton Mandeville*),
*Late Chief Medical Officer, Mission Hospital,*
*Kalna, Burdwan, Bengal.*

A FEW personal notes concerning a disease which may occasionally be met with in a shipping centre like Bristol may be of interest to some readers of this Journal.

To my mind there is scarcely a more dramatic proof of the advance of modern scientific medicine than the cure of this once dread disease.

I well remember that prior to the introduction of antimonial treatment we considered every case of kala azar practically hopeless—98 per cent. mortality. Now the majority of cases treated with antimony recover.

Kala azar is endemic in the Madras Presidency, Bengal, Assam and Bihar.

I took charge of the Mission Hospital at Kalna, Burdwan, Bengal, from 1922–24, in the centre of the endemic area, where my predecessor, Dr. Ernest Muir, had done his brilliant pioneer work in the treatment of this disease. During the last year of this period I treated some 400 cases at the Out-patient Department, and had about 150 cases in hospital. (The hospital has 64 beds.)

### Clinical Picture.

There is a marked wasting of the whole body, with the exception of the abdomen, which appears to enlarge as the rest of the body wastes, due to the steadily increasing size of the spleen and liver.

The hair becomes dry and scanty and falls out. The skin becomes shiny, acquires a darker hue in Indians, and appears to be tightly stretched over the bony parts. The darkening of the skin has led to the name "kala azar"—black fever—not to be confounded with black-water fever.

The large vessels in the neck throb from the low blood-pressure. The general appearance of any fairly advanced case of kala azar is typical, and is not likely to be mistaken for a case of malarial cachexia by anyone familiar with the two diseases.

The usual history is that a young adult or child (the ages most affected being three to thirty years) one and a half to three months after visiting an endemic area develops a fever often resembling typhoid in its initial ladder-like climb. The tongue, however, is clean, malaise is not marked and the mind is quite clear.

This initial fever of kala azar may resemble malaria, with sharp daily rigors, and a temperature running up to 103° or 104°.

After this initial flare-up, the fever often takes on a chronic low form for a few weeks, and then may show a rather striking peculiarity of the disease, namely a double rise of temperature, usually about 3.0 p.m. and at midnight.

In the early stages of the fever the unfortunate patient is usually drenched with quinine by his unsuspecting and well-meaning physician.

Untreated, the fever continues more or less all the time until death claims its victim in from three to twelve months.

*Spleen.*—This steadily increases in size as the disease lays hold of the patient. I have often seen cases where the abdomen is almost entirely filled by the enlarged spleen. After three months of the disease the spleen may be half-way down to the umbilicus, in six months at the umbilicus, in nine months almost down to the pubes. The acuter the disease the softer the spleen on palpation.

Leishman-Donovan bodies are usually found on spleen-puncture ; an operation not devoid of risk because the coagulability of blood is low in kala azar.

*Liver.*—This can nearly always be felt soft and sharp, perhaps two or three inches beneath the costal margin.

Leishman-Donovan bodies can be found in the liver on puncture, but not so easily as in the case of the spleen, though the risk of hemorrhage is less.

*Blood.*—Enthusiasts are, I believe, able to recover Leishman-Donovan bodies from the peripheral blood, but you need to be an enthusiast to achieve this. There is a very marked leucopenia, the white corpuscles being reduced to two or even one thousand. The deficiency is mainly due to a deficiency in polymorphonuclear leucocytes.

The coagulability of the blood is much decreased, so that bleeding from nose, gums, bowel and even stomach are very common. Petechiæ may occur in the skin.

The pulse is weak and rapid, the carotids pulsate, and the systolic blood-pressure may fall below 100 mm.

*Respiratory System.*—Owing to the defective defence offered by the polymorphs, inflammatory conditions of the lungs are very liable to occur, and death from broncho-pneumonia is one of the commonest terminations of the disease.

*Digestive System.*—The tongue is clean, the appetite quite good, but digestion is decidedly impaired. Diarrhœa and dysentery are very common. Needless to say, emetine is useless in dysentery due to kala azar.

In the final stages of the disease cancrum oris is very liable to occur and leads to death, from exhaustion in the majority of cases, but occasionally from hemorrhage. Still more rarely does spontaneous recovery take place from this most terrible complication. In such a case a leucocytosis occurs, and the result is not merely a checking of the facial necrosis, but a complete recovery from the disease. This happens in one or two per cent. of cases, the other 98 per cent. of cases being fatal if untreated.

### Diagnosis.

My facilities for laboratory work at Kalna were primitive in 1922, and I had no trained assistant at the time, so that I did not carry out spleen puncture and blood stainings, but of all the hundreds of cases I saw and treated I do not think a case was missed by doing Napier's aldehyde test and checking with the clinical symptoms already described.

This test was performed on about 1,000 cases. It is extremely simple, and consists of withdrawing about

5 c.c. of blood from a vein, allowing it to stand for a few hours in a test-tube, pouring off the separated serum and adding one drop of commercial formalin to each cubic centimetre of serum so collected. Shake and allow to stand for twenty-four hours. In a few moments the serum becomes viscid and gradually sets into a whitish opaque solid mass, like the hard-boiled white of an egg, if the case be one of kala azar of three or four months' duration. If the case is of less than three months' duration the serum may form a clear jelly-like mass in twenty-four hours, yellow or greenish in colour. This may indicate an early case of kala azar, chronic malaria, phthisis, leprosy or (as in one of my cases) actinomycosis.

If the serum remains perfectly liquid after twenty-four hours, the case is negative as far as kala azar is concerned. It should be noted that the serum remains strongly positive for some months after the disease is really cured, and slides from spleen-puncture are negative. Six months after cure the blood should be negative.

The disease most likely to be confounded with kala azar is malaria, but kala azar can usually be diagnosed in its early stages by a process of exclusion —absence of malaria parasites, non-reaction to quinine, and marked reaction to antimony injections.

### Treatment.

My routine treatment at Kalna was as follows :—

A two per cent. solution of sodium antimony tartrate in distilled water is boiled for five to ten minutes and 0·25 per cent. carbolic acid added if the solution is to be kept three or four days. It is, however, better made fresh as required.

This solution is injected intravenously three days a week, the initial dose being 0·5 c.c. for an adult and 0·5 c.c. increase for each succeeding dose until 5 c.c. is reached, the dose being then kept at that maximum figure until the case is considered cured. No increase should be made if there are rigors, vomiting, dizziness, much rise of temperature, pains in joints, or severe coughing. Children stand relatively large doses well.

It is often by no means an easy business to give an intravenous injection to a frightened, dark-skinned, wasted youngster of five or six years of age, with a low blood-pressure, and I have found that the veins on the back of the hand in a child are often easier to get into than the median-basilic.

There are one or two points to note about the injection :—

1. A single drop of the solution outside the vein will excite the most severe cellulitis, causing untold difficulty, only overcome by large bribes of sweets to persuade a child to submit to further treatment.

2. The solution must be injected extremely slowly, otherwise coughing, vomiting and faintness will be excited.

3. The injection is best given with the patient lying down, though I admit that most of mine were treated in the squatting position.

4. No food should be allowed for several hours before the injection, in order to avoid vomiting.

5. There is often a rise of temperature after each injection at first, until the disease begins to be "brought under."

*Duration of Treatment.*—My working rule was, roughly, that the duration of treatment should be proportionate to the time taken to bring down the fever, in the proportion of a week to a month, *e.g.* if the fever takes three weeks to come down to normal, then three months' treatment is indicated. In any case, no patient was discharged under two months, and one must take into consideration the amount of flesh put on, reduction of size of spleen, and absence of complications, etc.

*Subsidiary Treatment.*—Occasionally I used red oxide of mercury ointment to rub over the splenic area to excite leucocytosis, and also partly to please my patients, who hungered for "something to rub in." It seemed to me that I got as good results without the administration of painful irritants as with. Muir, however, is a great believer in intramuscular injections of creosote and turpentine "to excite leucocytosis." Digitalis is a useful drug to raise the blood-pressure, and was an ingredient in my stock iron and digitalis tonic.

I have tried other preparations of antimony intramuscularly in small children, but found them very painful, often causing abscesses. There have been scores of new preparations and remedies tried, but sodium antimony tartrate still holds the field. It is less toxic than potassium antimony tartrate, the first salt in use.

*The Diet* must be easily digested on account of liability to gastric disturbances.

*The Complications.* — Cancrum oris, dysentery, epistaxis, pneumonia, etc., must be treated symptomatically. Generally they call for a reduction of the dose of antimony for the time being, on account of the depressing effect of so powerful a remedy on an already badly debilitated subject, which might prove to be the "last straw."

The complications, on the other hand, if not too severe, may be actually helpful in producing a leucocytosis.

In conclusion, I must acknowledge my indebtedness to Dr. Ernest Muir, M.D., F.R.C.S., Leprosy Research Worker at the School of Tropical Medicine, from whom I have received much valuable help at various times in India in the study of kala azar.

## Figures and Tables

**Figure f1:**
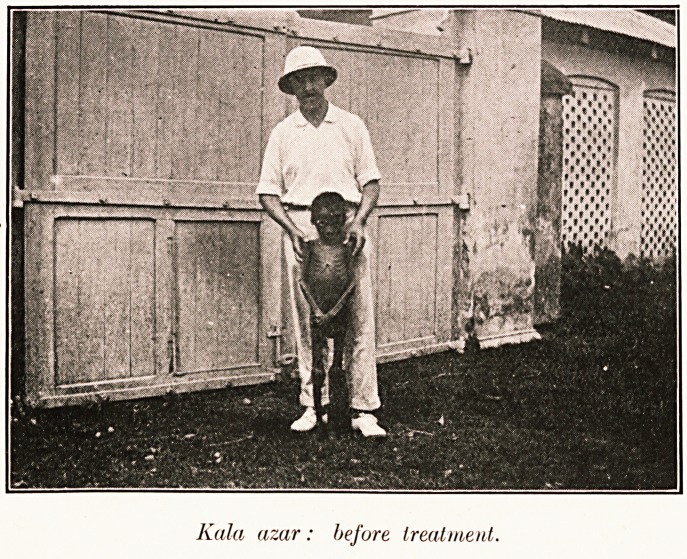


**Figure f2:**